# Correction: Monitoring of Cd and GSH contents and *Bn-OASTL* expression in transgenic tobacco seedlings in response to Cd stress

**DOI:** 10.1371/journal.pone.0345957

**Published:** 2026-03-25

**Authors:** Xiaolan He, Jianwei Wang, Wenxu Li, Xinhong Chen

There are number of errors in Fig 1 and 2. Please see the correct Fig 1 and 2 here.

**Fig 1:**
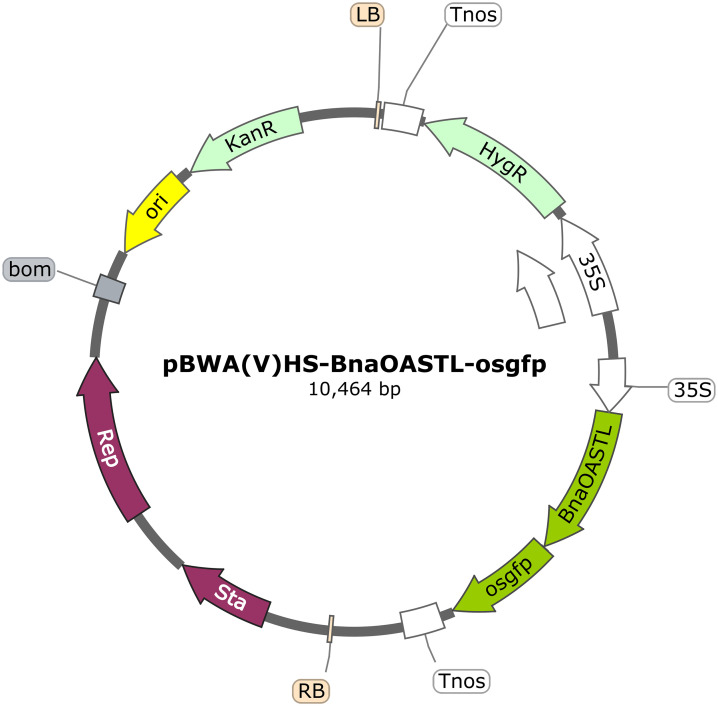
Schematic of the recombinant vector. RB and LB, right and left T-DNA borders; 35S, CaMV 35S poly A; HYG, hygromycin B; osgfp, the BnaOASTL-GFP fusion gene; Tnos, nopaline synthase terminator.

**Fig 2:**
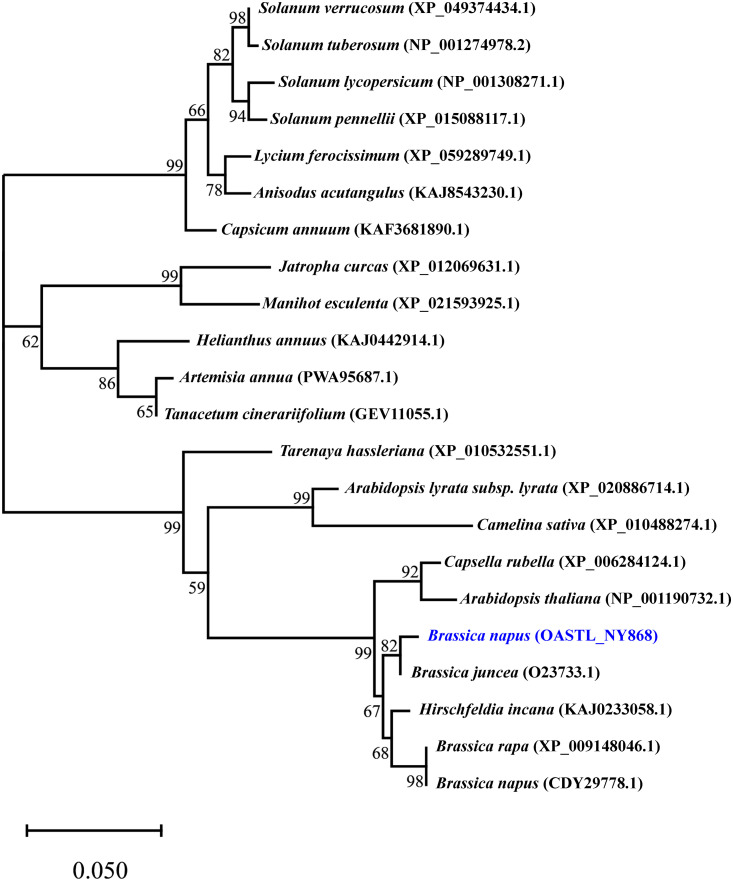
Phylogenetic tree of OASTL proteins from B. napus and other species based on amino acid sequences. Bootstrap values (>50%) from 1000 replicates are demonstrated at branch nodes. The scale bar represents a distance of 0.05. The B. napus OASTL sequence is marked with a blue symbol. Other sequences were obtained from XP_049374434.1, NP_001274978.2, NP_001308271.1, XP_015088117.1, XP_059289749.1, KAJ8543230.1, KAF3681890.1, PWA95687.1, GEV11055.1, KAJ0442914.1, XP_012069631.1, XP_021593925.1, OASTL_NY868, O23733.1, XP_009148046.1, CDY29778.1, KAJ0233058.1, XP_006284124.1, NP_001190732.1, XP_020886714.1, XP_010488274.1 and XP_010532551.1.
