# Supplementary figures and images for: Monitoring of Cd and GSH contents and Bn-OASTL expression in transgenic tobacco seedlings in response to Cd stress
Source: PLoS One. 2026 Jan 22;21(1):e0329885. doi: 10.1371/journal.pone.0329885 (PMC12826462; doi:10.1371/journal.pone.0329885)

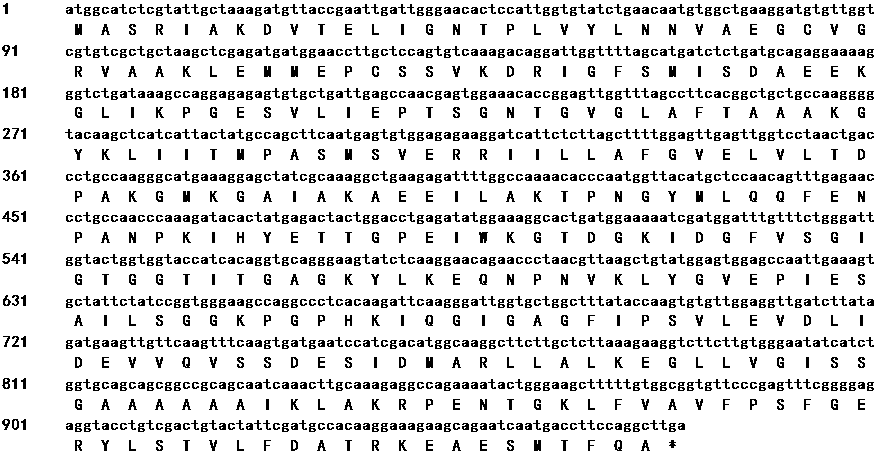

Supplement: S1 Fig — (TIF) [file pone.0329885.s001.tif]

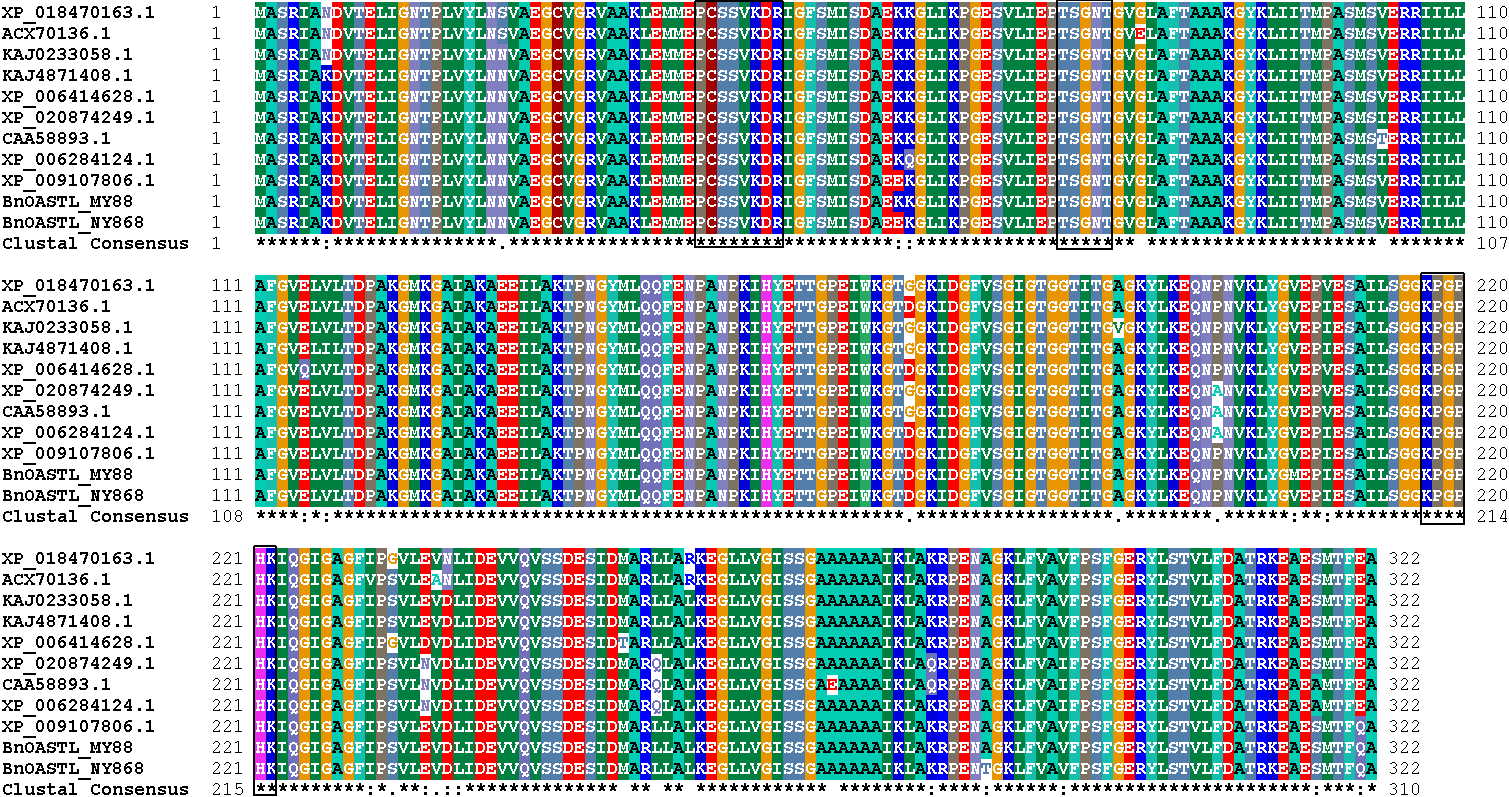

Supplement: S2 Fig — Identical residues are marked with asterisks, conserved substitutions with colons, and semiconserved substitutions with periods. The conserved domains: TSGNT (substrate-binding site), KPGPHK (SAT1-binding site), and PXXSVKDR (PLP-binding site), are boxed. The BnaOASTL sequence was obtained from B. napus, and other sequences from XP_018470163.1, ACX70136.1, KAJ0233058.1, KAJ4871408.1, XP_006414628.1, XP_020874249.1, CAA58893.1, XP_006284124.1, and XP_009107806.1. (TIF) [file pone.0329885.s002.tif]
