# Supplementary material for: Monitoring of Cd and GSH contents and Bn-OASTL expression in transgenic tobacco seedlings in response to Cd stress
Source: PLoS One. 2026 Jan 22;21(1):e0329885. doi: 10.1371/journal.pone.0329885 (PMC12826462; doi:10.1371/journal.pone.0329885)
Supplement: S1 Raw image — (PDF) [file pone.0329885.s003.pdf]

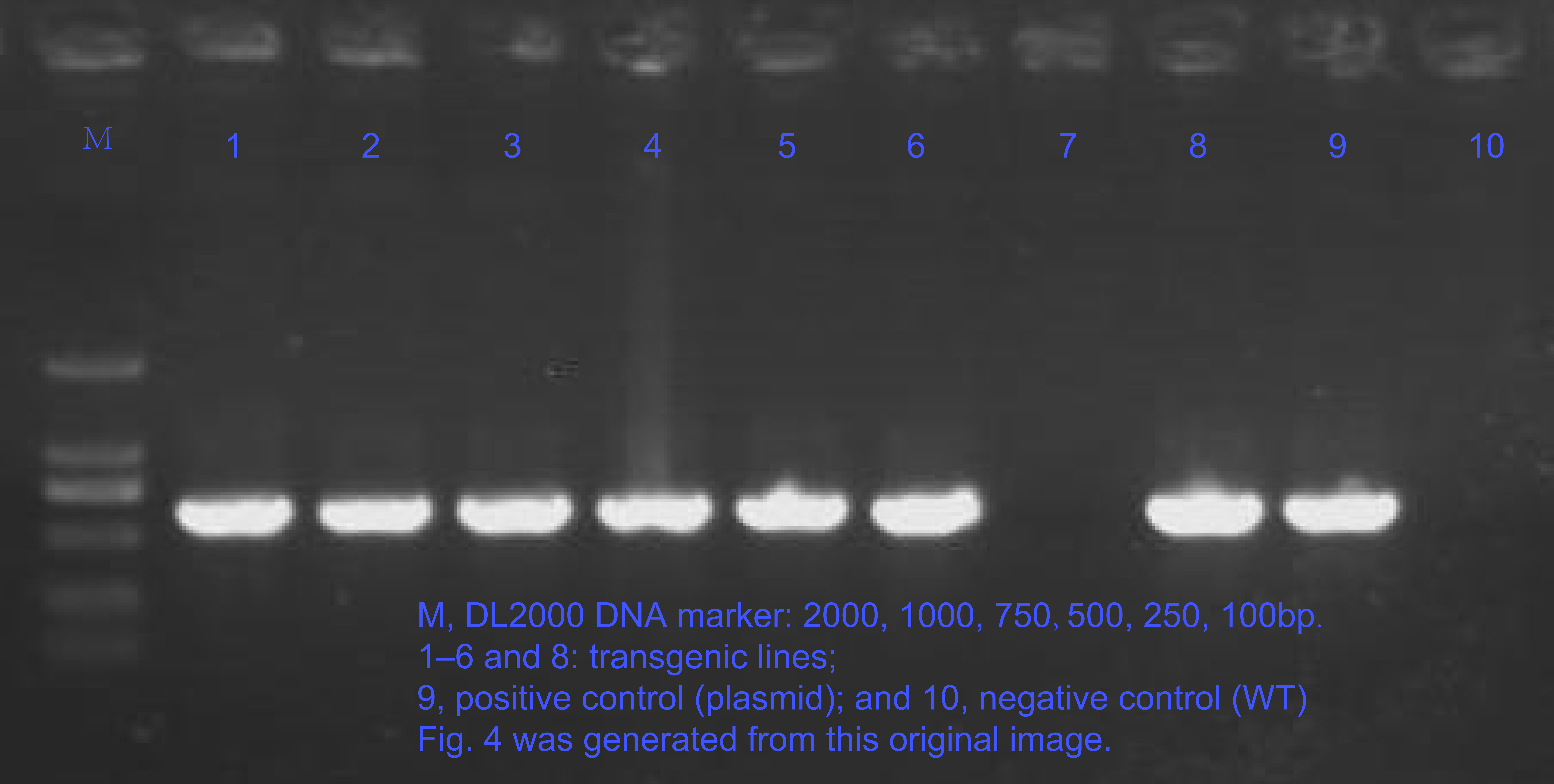
M
1
2
3
4
5
6
7
8
9
10
M, DL2000 DNA marker: 2000, 1000, 750, 500, 250, 100bp.
1–6 and 8: transgenic lines;
9, positive control (plasmid); and 10, negative control (WT)
Fig. 4 was generated from this original image.
