# Supplementary material for: Monitoring of Cd and GSH contents and Bn-OASTL expression in transgenic tobacco seedlings in response to Cd stress
Source: PLoS One. 2026 Jan 22;21(1):e0329885. doi: 10.1371/journal.pone.0329885 (PMC12826462; doi:10.1371/journal.pone.0329885)
Supplement: Attachment — Submitted filename: Response to Editor and Reviewers-20250924.docx [file pone.0329885.s008.docx]

List of Responses

Dear Editors and Reviewers,

Thank you for your letter and for the reviewers’ constructive comments on our manuscript entitled “Physiological and *Bn-OASTL* Gene Expression Responses to Cadmium Stresses in Tobacco Seedlings” (Manuscript ID:PONE-D-25-39819). The comments have been highly valuable in helping us revise and improve the paper, and they also offer important guidance for our future research. We have carefully considered each comment and made corresponding corrections, which we hope will meet with your approval. All revisions are marked in red in the revised manuscript. Below, we provide a point-by-point response to the comments.

Responses to the Editor and Reviewers’ Comments:

Editor’s Comments:

1.We noted some minor overlap with the following previous publications, which needs to be addressed:

https://pubmed.ncbi.nlm.nih.gov/38775862/

https://www.mdpi.com/2311-7524/8/11/1002

Response: Thank you for bringing this to our attention. We have carefully reviewed the text and revised the manuscript prior to resubmission to ensure all overlapping content has been properly quoted or paraphrased, with appropriate citations included.

2.In your revision, ensure you cite all your sources (including your own works), and quote or rephrase any duplicated text outside the methods section. Further consideration is dependent on these concerns being addressed.

Response: We sincerely thank you for this reminder. We have now ensured that: All sources, including our own previous works, are properly cited. Any text outside the Methods section that resembled previously published content has been either quoted with citation or rephrased to ensure originality. A thorough check has been conducted to confirm proper attribution and originality throughout the manuscript.

Reviewer #1:

1.The title, abstract, and keywords are not fully consistent regarding the research subject, species, and gene information. For example, the title and abstract refer to Bn-OASTL and tobacco, while the keywords include "rapeseed (Brassica napus L.)" instead. Please ensure that these elements are precisely aligned and consistently reflect the main experimental system and research focus.

Response: We thank the reviewer for this important observation. We have revised the title, abstract, and keywords to ensure they are consistent and accurately reflect the focus on *Bn-OASTL* and tobacco.

2.The abstract contains some ambiguous statements and lacks clear logical flow. In particular, the functional analysis of the gene did not show a significant effect in transgenic tobacco, but the discussion on the underlying mechanisms is insufficient. Furthermore, the scientific significance and future perspective of this study in the context of Cd-tolerant crop molecular design are not adequately highlighted. Please refine the abstract to clearly state the main findings (e.g., the lack of improvement in Cd tolerance/accumulation) and emphasize their scientific relevance to the field.

Response: We appreciate the reviewer’s insightful suggestions. The abstract has been thoroughly revised to improve clarity and logical flow. We now clearly state the main finding—that overexpression of Bn-OASTL did not enhance Cd tolerance or accumulation—and discuss possible reasons and the broader implications for metabolic engineering and phytoremediation.

3.The introduction is overly lengthy and the literature review is somewhat scattered. I recommend condensing the introduction to make it more concise, with a clear focus on the scientific question and the innovative aspects of this study. Excessive background information should be removed to avoid redundancy. Please clearly articulate the rationale for selecting BnOASTL, the reason for using tobacco as the heterologous expression system, and the current knowledge gaps that this work aims to address. In addition, the introduction should include a more forward-looking discussion on the potential of the OASTL gene family in molecular breeding and heavy metal phytoremediation, highlighting future directions and scientific significance.

Response: We agree with the reviewer’s suggestion. The introduction has been significantly condensed and refocused to clearly present the rationale for selecting BnOASTL, the use of tobacco as an expression system, and the knowledge gaps this study addresses. We have also added a forward-looking perspective on the role of OASTL genes in molecular breeding and phytoremediation.

4.Provide more experimental details, such as specific reagent sources, PCR/qPCR conditions, and statistical methods to improve reproducibility. Ensure all primers, vectors, and key reagents are clearly described.

Response: We thank the reviewer for this suggestion. The Materials and Methods section has been expanded to include detailed information on reagent sources, PCR/qPCR conditions, primer sequences, vectors, and statistical analyses to enhance reproducibility.

5.Enhance the interpretation of the results, especially the possible reasons why overexpression of BnOASTL did not improve Cd tolerance. Discuss the broader significance and possible directions for future research, such as the need for multi-gene manipulation or systems biology approaches.

Response: We are grateful for this constructive comment. The Discussion section has been substantially expanded to interpret the lack of phenotypic improvement, including possible reasons such as metabolic bottlenecks, feedback regulation, and subcellular compartmentalization. We also discuss the broader implications and propose future directions, including multi-gene stacking, systems biology approaches, and the use of stress-inducible promoters.

6.Ensure that all figures and legends are sufficiently detailed, and clearly indicate statistical significance where applicable.

Response: We have revised all figures and legends to include necessary details and have clearly marked statistical significance where relevant.

7.Please ensure that all references are properly formatted and include DOI numbers where available. Including DOIs for all cited literature is required by the journal and will improve the accessibility and traceability of your references.

Response: We have carefully checked and reformatted all references according to the journal’s guidelines, adding DOIs where available to improve accessibility and traceability.

Reviewer #2:

1.The title did not match the manuscript. There is no physiological study; it is just a monitoring of Cd and GSH contents.

Response: We apologize for this oversight. The title has been revised to: “Monitoring of Cd and GSH Contents and Bn-OASTL Gene Expression in Response to Cadmium Stress in Tobacco Seedlings.”

2.In the manuscript, authors present a work on tobacco plants; however, in the conclusion they change to tomato???

Response: We sincerely apologize for this error. The conclusion has been corrected to refer to tobacco throughout.

3.Molecular analyses are good; however, the discussion is not appropriate to the found results. It is clear from results that the Bn-OASTL gene has no relationship with the resistance behavior within tobacco plant, meaning that tobacco did not use the O-acetylserine(thiol)lyase pathway to overcome the Cd stress.

Response: We thank the reviewer for this critical observation. The Discussion has been revised to better align with the results. We now emphasize that Bn-OASTL overexpression did not enhance Cd tolerance in tobacco, suggesting that this pathway may not be central to Cd detoxification in this species. We also discuss alternative mechanisms and the need for further research to understand Cd response in plants.

We are sincerely grateful to the editors and reviewers for their valuable comments, which have greatly improved the quality of our manuscript. We hope that the revised version is now suitable for publication.

Thank you for your time and consideration.

Yours sincerely,

Jianwei Wang, Ph.D.

Professor

School of Science, Kaili University

Kaili, 556011, GuiZhou, China

Email: agan1982@126.com

September 22, 2025
